# MXene/Graphene Oxide Heterojunction as a Saturable Absorber for Passively Q-Switched Solid-State Pulse Lasers

**DOI:** 10.3390/nano11030720

**Published:** 2021-03-12

**Authors:** Yunjia Wang, Jianwen Wang, Qiao Wen

**Affiliations:** Key Laboratory of Optoelectronic Devices and Systems of Ministry of Education and Guangdong Province, College of Physics and Optoelectronic Engineering, Shenzhen University, Shenzhen 518060, China; wangyunjia@szu.edu.cn (Y.W.); wangjianwen2018@email.szu.edu.cn (J.W.)

**Keywords:** MXene, saturable absorber, heterojunction, Q-switched, pulsed laser, nonlinear optics, bulk laser

## Abstract

Owing to their unique characteristics, two-dimensional (2-D) materials and their complexes have become very attractive in photoelectric applications. Two-dimensional heterojunctions, as novel 2-D complex materials, have drawn much attention in recent years. Herein, we propose a 2-D heterojunction composed of MXene (Ti_2_CT*_x_*) materials and graphene oxide (GO), and apply it to an Nd:YAG solid-state laser as a saturable absorber (SA) for passive Q-switching. Our results suggest that a nano-heterojunction between MXene and GO was achieved based on morphological characterization, and the advantages of a broadband response, higher stability in GO, and strong interaction with light waves in MXene could be combined. In the passively Q-switched laser study, the single-pulse energy was measured to be approximately 0.79 µJ when the pump power was 3.72 W, and the corresponding peak power was approximately 7.25 W. In addition, the generation of a stable ultrashort pulse down to 109 ns was demonstrated, which is the narrowest pulse among Q-switched solid-state lasers using a 2-D heterojunction SA. Our work indicates that the MXene–GO nano-heterojunction could operate as a promising SA for ultrafast systems with ultrahigh pulse energy and ultranarrow pulse duration. We believe that this work opens up a new approach to designing 2-D heterojunctions and provides insight into the formation of new 2-D materials with desirable photonic properties.

## 1. Introduction

In recent years, two-dimensional (2-D) materials, including semiconductors, transition metal dichalcogenides, and topological insulators, have been widely applied in the photoelectric and biomedical fields due to their unique structures [1,2,3,4,5]. Two-dimensional heterojunctions, as novel 2-D materials constructed by combining different 2-D materials, were demonstrated to possess a broad optical response, a tunable band gap, and strong interaction with photons [6,7,8,9,10,11,12]. Thus, the advantages of the different 2-D materials were expected to be combined in such 2-D heterojunctions when incident light passed through the heterojunction boundary, obtaining an optimum photoelectric performance [13]. Due to these excellent characteristics, the use of 2-D heterojunctions in photoelectric devices has been reported, such as in solar cells, optical communication devices, and photodetectors [14,15,16,17,18,19,20,21]. Owing to these significant studies, the applications of 2-D materials have been rapidly broadened, accompanied by fast development in their photoelectric applications.

For example, solar cells using 2-D heterojunctions have been reported, obtaining better absorption coefficients and excellent stability [22,23,24,25]. In addition, mid-infrared detectors employing 2-D heterojunctions were also reported to achieve excellent performance due to the optimum structure of heterojunctions [26,27,28]. However, the preparation conditions of such 2-D heterojunctions were strict, and their application mechanism has not been clarified. In the past several years, 2-D heterojunctions as saturable absorbers (SAs) in laser technology have also been reported due to their ideal nonlinear optics [29,30,31]. Our team has reported the fabrication of 2-D graphene/phosphorene (BP) nano-heterojunction-based optical SAs, which showed excellent performance in an erbium-doped fiber laser, demonstrating the generation of a stable ultrafast pulse down to 148 fs [32]. Notably, nanosized BP was unstable under ambient conditions, so more protection was needed to eliminate oxidation. In addition to the synthesis of graphene/phosphorene (BP) nano-heterojunction SAs, a graphene/Bi_2_Te_3_ 2-D heterojunction SA was proposed in recent years and achieved excellent performance in mode-locking operation and Q-switched operation [33]. These results indicate that desirable photonic properties could be obtained by employing a 2-D heterojunction; however, few studies on MXene-based 2-D heterojunctions as SAs applied in passively Q-switched solid-state lasers have been reported, and the mechanism in passively Q-switched solid-state lasers is still unknown.

Herein, we propose a facile way to synthesize a MXene–graphene oxide (MXene–GO) 2-D heterojunction in a liquid phase, and a passively Q-switched laser in the solid-state using the MXene–GO 2-D heterojunction as an SA is investigated. Here, few-layer graphene oxide (GO) chosen to form 2-D heterojunctions with MXene materials was chosen not only for its higher damage threshold, lower saturation intensity, and broad absorption band, but also expected to tune the optical properties of MXene and improve its performance in optoelectronics [34,35,36,37]. Our results suggest that the MXene–GO 2-D heterojunction has better performance than pristine graphene and pure MXene. In passive Q-switching operation, we obtained a stable ultrashort pulse down to 109 ns, which is the narrowest pulse among Q-switched lasers in the solid-state using a 2-D heterojunction as an SA. We believe that our work paves the way to designing MXene-based materials to obtain passively Q-switched lasers in the solid state, and indicates that the MXene–GO composite exhibits higher performance in nonlinear optics and acts as a potential 2-D material for photoelectric applications.

## 2. Experimental

### 2.1. Fabrication of the MXene–GO SA

GO solution dispersed in ionic water at 0.5 mg/mL was obtained from a commercial supplier (Nanotechnology, Beijing, China), and MXene (Ti_2_C) (99.9%) powder and isopropanol (IPA) solvent were supplied by Aladdin Co., Ltd. (Shanghai, China). All the other reagents and solvents were supplied from commercial sources and used without further purification unless otherwise noted. The mixture of MXene and GO, which both had a few-layer structure, was reacted in a liquid phase via chemical bonding or the Coulomb force. In brief, 5 mL GO solution at 0.5 mg/mL was put into 3 mL IPA solvent to achieve a uniform dispersion liquid. After that, GO with a few-layer structure was obtained via centrifugation at 1000 rpm for 5 min. Next, 8 mg multilayer MXene powder was put into IPA solvent and sonicated in an ultrasonic bath continuously for 10 min to achieve a dispersion liquid. Next, the dispersion liquid was centrifuged at 8000 rpm for 5 min to achieve few-layer MXene. Finally, a mixture of MXene and GO was prepared by combining the mother liquids in a volume ratio of 3:2, at a pH of 4, and fully magnetically stirring them for 1 h at room temperature.

**Figure 1:**
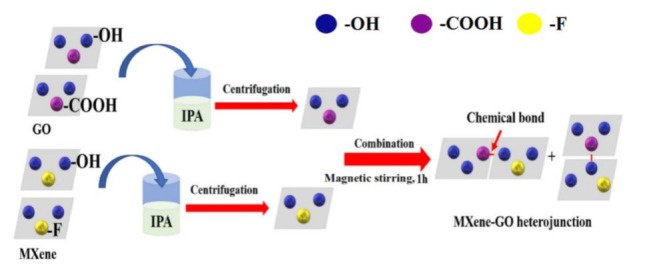
Illustration of the MXene–graphene oxide (MXene–GO) heterojunction preparation process.

An illustration of the preparation scheme is shown in Figure 1. As depicted, the blue balls represent the -OH group, the purple balls in the image represent the -COOH group of the GO material, and the yellow balls are the -F group on the surface of the MXene material. Thanks to these hydrophilic groups, the GO and MXene materials can be contacted easily in the IPA solvent, interaction would take place after the combination, and a chemical bond is formed under the magnetic string for 1 h.

### 2.2. Characterization of the MXene–GO SA

After the reaction, the MXene–GO sample was transferred onto a silica substrate for morphological characterization by scanning electron microscopy (SEM, Hitachi, SU8010, Tokyo, Japan), and elemental mapping was conducted by using energy dispersive spectroscopy (EDS). After centrifugation at 10,000 rpm for 5 min, the sediment-containing MXene–GO mixture was dispersed in absolute ethyl alcohol and then dropped onto a silica substrate for structural characterization and selective area electron diffraction (SAED), by using high-resolution transmission electron microscopy (HR-TEM, FEI, Tecnai G2 F30, Zhenzhou, China). After that, the sample was transferred onto a carbon film copper grid for thickness measurement, by using atomic force microscopy (AFM, Bruker Dimension Icon, Beijing, China). Raman spectra were obtained on a Raman spectrometer (Renishaw in Via Reflex, Shanghai, China) at room temperature for further analysis of the sediment structure. Absorption measurements were performed to analyze the optical properties by using a spectrophotometer (Agilent Cary 5000, Jiangxi, China) at room temperature.

**Figure 2:**
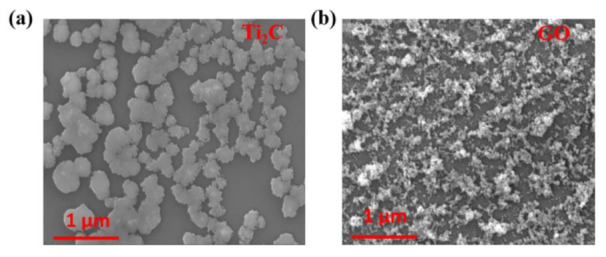
(**a**) Scanning electron microscopy (SEM) image of MXene (Ti_2_C) material; (**b**) SEM image of graphene oxide (GO) material.

Figure 2 shows the morphological characterization of the prepared MXene and GO materials via SEM. As depicted in Figure 2a, the MXene materials showed sheet structure with sub-micro size according to the scale bar in the SEM image, while the GO materials exhibited a cluster-like shape, which is displayed in Figure 2b.

**Figure 3:**
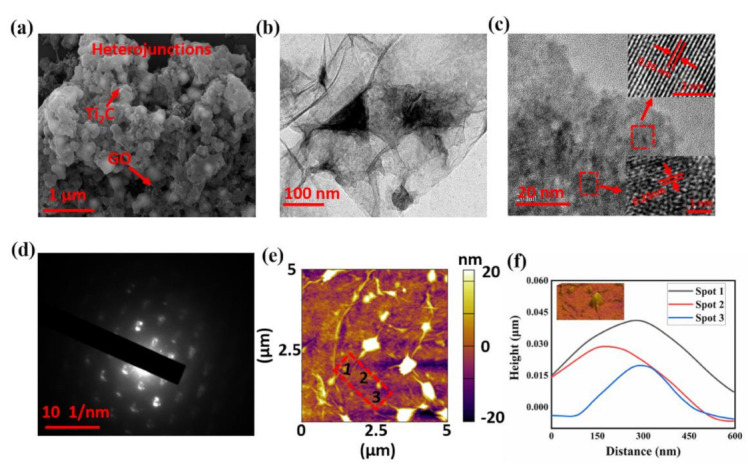
Morphological and structural characterization of the heterojunction: (**a**) SEM image of the heterojunction; (**b**) TEM image of the heterojunction; (**c**) high-resolution transmission electron microscopy (HR-TEM) image of the heterojunction: top right corner and bottom right corner show two different lattice distances; (**d**) selective area electron diffraction (SAED) pattern of the heterojunction; (**e**) atomic force microscopy (AFM) image of the heterojunction; (**f**) corresponding height profiles of the heterojunction.

Figure 3 shows the morphological and structural characterization of the heterojunction. As depicted in Figure 3a, the heterojunctions showed a sheet structure with sub-micro size, while the TEM result displayed in Figure 3b reveals that the heterojunction consisted of two different structures. Deep structure analysis of this heterojunction is shown by HR-TEM in Figure 3c. Two different lattice distances were present in the top right corner and bottom right corner of the image, which were measured as 0.23 and 0.36 nm, respectively. The value difference was beyond the resolution of the equipment we applied. Thus, we inferred that the two different lattice distances originated from two different structures. Figure 3d shows the SAED image obtained from the rectangular region in Figure 3b which exhibits two different diffraction spots of both heterojunctions. All the above analyses suggest that the MXene sheets adhered to other sheets, and a phase boundary was observed of the mixture. The bright spots in the AFM image shown in Figure 3e further confirm the phase boundary of the heterojunction, and the height profiles of the heterojunction shown in Figure 3f were obtained from three spots in Figure 3e, which reveal that the thickness of the heterojunction was between 15 and 25 nm.

**Figure 4:**
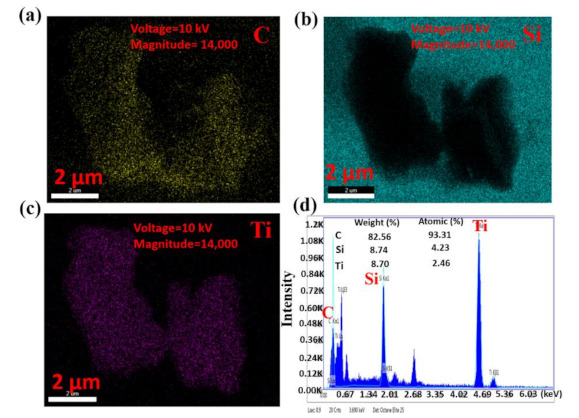
Elemental mapping and optical properties of the MXene–GO heterojunction: (**a**) carbon, (**b**) silica, and (**c**) titanium elemental analysis of the MXene–GO heterojunction; (**d**) element ratios of the MXene–GO heterojunction analyzed by energy dispersive spectroscopy (EDS).

Elemental mappings of the heterojunction obtained by EDS were used to further confirm the composition of the heterojunctions, which are shown in Figure 4a–c. As depicted, titanium and carbon were both found in the heterojunction, while silica was also exhibited in the EDS spectrum because it was used as a substrate. Figure 4d presents the different elemental analyses of the MXene–GO material by weight ratio and atomic ratio. As the measurement results show, the weight ratios of carbon, silica, and titanium were 82.56%, 8.74%, and 8.70%, respectively, while the atomic ratios were 93.31%, 4.23%, and 2.46%, respectively. The much higher weight ratio and atomic ratio of carbon than of titanium suggest that collective -COOH groups were fixed on the surface of GO, attributed to the convenient effect of the MXene material and GO in forming the MXene–GO heterojunction.

**Figure 5:**
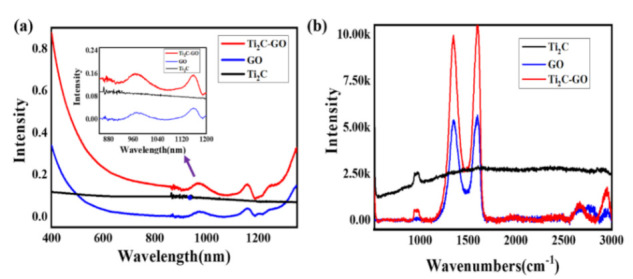
(**a**) Absorption spectra of the MXene–GO heterojunction, MXene material, and GO material; (**b**) Raman spectra of the MXene–GO heterojunction, MXene material, and GO material.

Figure 5a shows the absorption spectrum of the MXene–GO heterojunction, as well as the spectra of the MXene material and GO material for comparison. Here, we kept the same concentration of pure MXene, pure GO, and the MXene–GO heterojunction and measured them in the liquid phase. As depicted, the MXene material and GO material both have a broadband optical response, and the MXene–GO heterojunction takes full advantage of these two materials, resulting in a higher linear absorption intensity. Figure 5b presents the Raman spectrum of the MXene–GO heterojunction at room temperature to further demonstrate the formation of the MXene–GO heterojunction. The spectra of the MXene material and GO material were both measured for comparison.

As depicted, the Raman peak located at approximately 1000 cm^−1^ was ascribed to the Raman mode of the Ti_2_C material, and the Raman peaks located at approximately 1350 and 1600 cm^−1^ were ascribed to the Raman mode of the GO material, which coincided with the literature [38,39,40]. However, when the MXene and GO materials were combined, Raman peaks of 1000, 1350, and 1600 cm^−1^ were found in the Raman spectra of the mixture, and the intensity of the Raman peaks was modified: for example, the Raman peak located around 1000 cm^−1^ was lower than the pure Ti_2_C materials, while the Raman peaks located around 1350 and 1600 cm^−1^ were both higher than the pure GO material. Hence, we inferred that the MXene materials may be oxidized to TiO_2_, and thus the combination of MXene and GO materials would result in the vibration of Raman peaks via the affection of GO materials and TiO_2_, which has been publicly reported in previous work [38,41]. However, more experiments would be needed to further demonstrate that indication.

**Figure 6:**
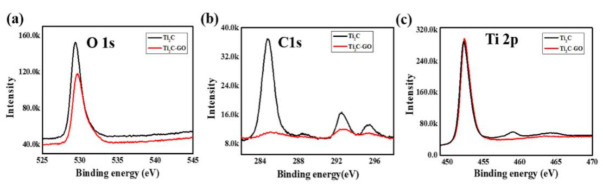
X-ray photoelectron spectroscopy (XPS) characterization of MXene and MXene–GO materials: (**a**) oxide element analysis; (**b**) carbon element analysis; (**c**) titanium element analysis.

Figure 6 shows the X-ray photoelectron spectroscopy (XPS) characterization of MXene and MXene–GO materials. The peaks of O 1s, C 1s, and Ti 2p are, respectively, exhibited in Figure 6a–c, which can be matched to the results of EDS. As is depicted in Figure 6a, the peak of the oxide element for MXene materials was decreased when combined with GO materials. A similar phenomenon can be found for the carbon element, shown in Figure 6b. However, the peaks around 459 and 465 eV for the titanium element of MXene materials disappeared when the combination of MXene materials and GO materials was achieved. We suggest the reason to be that part of the Ti–C bond was broken, and a new chemical bond was formed, which may have originated from the formation of the MXene–GO heterojunction [42].

To the best of our knowledge, the oxidation of the MXene material in the air leads to a decrease in the Raman signal intensity or the modification of some Raman modes [43,44,45]. This oxidation directly affects the long-term stability of the MXene material and restricts its broad applications. To further investigate the stability of the MXene material when combined with GO material, it was exposed to air for one week, and pure MXene material was used as a comparison. In situ measurements were conducted on these two samples.

**Figure 7:**
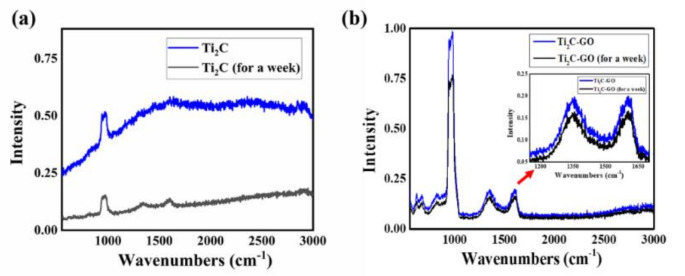
Stability test of the MXene–GO heterojunction: (**a**) Raman spectra of pure MXene (Ti_2_C) exposed to air for 0 days or a week; (**b**) Raman spectra of the pure MXene–GO heterojunction exposed to air for 0 days or a week.

Figure 7a shows the Raman spectra of pure MXene material, which displayed a remarkable decrease after exposure to air for 7 days. For convenient comparison, we assumed the Raman intensity after exposure to air for 0 days was I_1_, and after exposure to air for 7 days was I_2_. The stability factor can be presented as A = I_2_/I_1_. After the software analysis, we calculated that the scaling factor of pure MXene was 0.3, while the difference between the two Raman spectra of MXene–GO materials is displayed in Figure 7b. 

As depicted, the decreasing signal around 1000 cm^−1^ has a scale factor of 0.78, calculated by the above expression, which is higher than the scale factor of pure MXene in Figure 7a, and it should be noted that the decreasing signals around 1350 and 1600 cm^−1^ have a scale factor of 0.83, which is higher than the peak around 1000 cm^−1^. To the best of our knowledge, the Raman signal of the same sample may be changed at different spots due to the different concentrations of materials. To validate the comparison results, we observed the Raman signal of the same MXene material at different spots and found that the vibration was much smaller than that of the sample that was exposed to air for a week. Therefore, we inferred that these modifications did not originate from the different spots of the samples [43,44,45,46]. Thus, we inferred that the MXene materials were endowed with increased stability through combination with the GO materials. We believe that this work provides new insight that will help to improve the stability of MXene materials, to develop desired photoelectric applications.

### 2.3. Q-Switched Laser Setup

**Figure 8:**
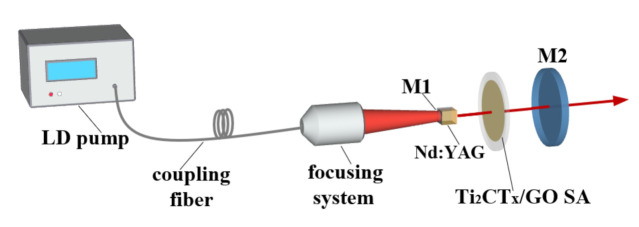
Schematic of the passively Q-switched Nd:YAG laser with a Ti_2_CT_x_-GO saturable absorber (SA).

To examine the nonlinear optics of the MXene–GO material as an SA, its performance in Q-switched laser application was examined. The laser resonator is shown in Figure 8. The pump was a semiconductor laser with a maximum output power of 30 W, and the center wavelength of the pump laser was 808 nm at 25 °C. The fiber numerical aperture was 0.22, and the core diameter was 200 microns. To obtain the appropriate size of the pump light on the laser crystal, we used a fiber output focusing lens with a 1:0.8 image ratio. The gain medium in the laser was a 3 mm × 3 mm × 4 mm Nd:YAG crystal with a 1.2 wt% doping concentration, which was cut at the (111) crystal face. In the experiment, we employed a short resonant cavity to generate the laser. Therefore, the S1 surface of the laser crystal coated with an 808 nm antireflection and a 1064 nm high-reflection coating served as the plane mirror part of the resonant cavity. The S2 surface was coated with an 808 and 1064 nm antireflection coating to obtain better pumping efficiency.

To ensure a good heat dissipation performance of the laser crystal, we wrapped the Nd:YAG crystal with indium foil, coated this foil with thermal grease, and finally placed the crystal on a copper base with a water cooling function, whose temperature was set at 17 °C. In the experiment, an output mirror with a curvature radius of 50 mm and a transmittance of 15% was used. The output mirror surface was coated with a coating that possessed high reflection at 808 nm and 15% transmittance at 1064 nm. With this designed coating, a relatively short plane-concave resonator of nearly 10 mm was formed. The MXene–GO heterojunction acted as an SA and was inserted into the laser resonator in the transmission path. By using the calculation of the ABCD matrix, we obtained a laser spot with a width of 88 microns on the SA. We supposed that a passively Q-switched pulsed laser would be achieved via such a small laser spot and that nonlinear effects may be produced by the MXene–GO SA. In the experiment, an optical power meter (30A-P-17, Ophir Optronics Solutions Ltd., Jerusalem, Israel) was used to measure the laser output power, and the laser output spectrum was obtained by applying an Ocean Optics spectrometer (USB4000-VIS-NIR, Ocean Optics Inc., Dunedin, EL, USA). In addition, the generation of a laser pulse was detected by an InAsSb photoelectric probe (DET10A/M, Thorlabs, Inc., Newton, NJ, USA), and the laser pulse generated by passive Q-switching was recorded by a high-speed digital oscilloscope (DPO4104B, Tektronix, Inc., Shawnee Mission, KS, USA) with a bandwidth of 1 GHz and a sampling rate of 5 GHz.

## 3. Results and Discussion

**Figure 9:**
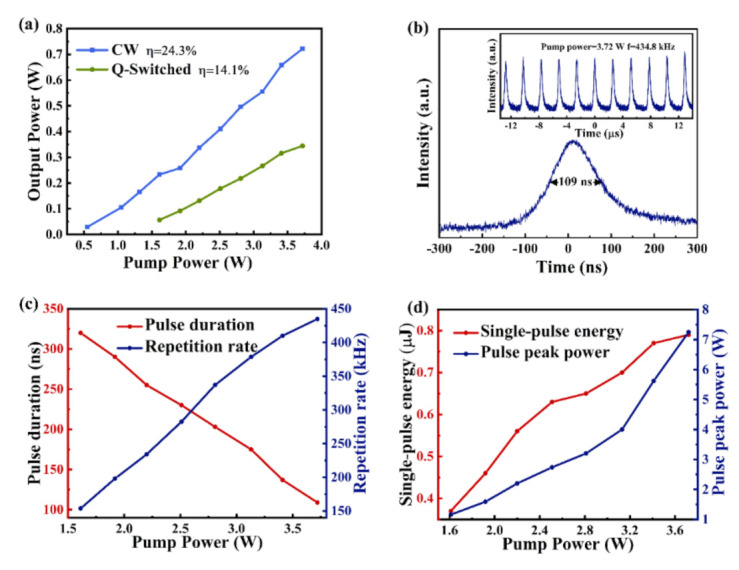
Q-Switched pulse output characteristics with the Ti_2_CT_x_-GO SA. (**a**) Average output power of continuous wave (CW) and Q-switched lasers under different pump powers; (**b**) single-pulse profile at a 3.72 W pump power with an output coupling (OC) mirror transmission of 15%; (**c**) evolution of the repetition rate and pulse duration with pump power; (**d**) single-pulse energy and peak power as a function of pump power.

To analyze the Q-switched laser, the output power under continuous operation of the Nd:YAG laser should be studied as a comparison. Figure 9a presents the variation in the average output power of the laser in passively Q-switched mode and compares it with that in continuous wave (CW) mode. When the transmission rate of the output coupling (OC) mirror was 15%, the corresponding pumping threshold was 0.55 W. When the pump power was increased to 3.72 W, a continuous laser output of 0.74 W could be obtained, and the corresponding slope efficiency was 24.3%. When the MXene–GO material was inserted into the resonator as an SA, a stable passively Q-switched pulse was obtained with a pump power of 1.61 W. As the pump power was increased to 3.72 W, the corresponding maximum output power of the passively Q-switched laser reached 344.2 mW, the corresponding slope efficiency was 14.1%, and the optical-optical conversion efficiency was 9.3%.

Figure 9b shows the corresponding shortest pulse width when the pump power was 3.72 W obtained from the oscilloscope; a stable ultrashort pulse down to 109 ns was achieved by the Nd:YAG solid-state laser via the MXene–GO heterojunction SA. Figure 9c depicts the variation in the pulse width and repetition frequency with pump power. With increasing pump power, the pulse width changed from 320 to 109 ns, and it presented a decreasing tendency, whereas an increasing trend was found for the repetition frequency, which changed from 153.8 to 434.8 kHz. When the pump power exceeded 3.72 W, the passively Q-switched pulse became unstable. When the pump power increases, the saturable absorber may have more heat accumulation, which leads to the structural destruction of the MXene–GO heterojunction SA. Therefore, we will consider coating the MXene–GO heterojunction SA with an antireflection coating to reduce the accumulation of heat on the SA, to increase the thermal damage threshold of the SA and conduct experimental studies related to high-power lasers [47,48]. Figure 9d shows the variation in the single-pulse energy and passively Q-switched pulse peak power with pump power. The single-pulse energy and pulse peak power both increased with increasing pump power. Notably, the single-pulse energy was 0.79 µJ when the pump power was 3.72 W, and the corresponding peak power was 7.25 W.

**Figure 10:**
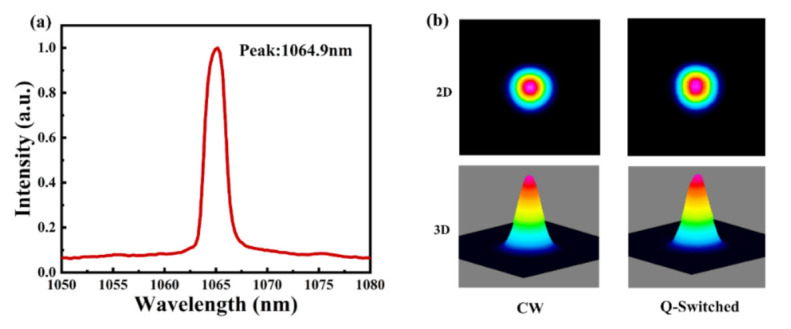
(**a**) Emission spectrum of the Q-switched laser; (**b**) beam profile of the laser in CW and Q-switched modes.

Figure 10a presents the output spectrum of the laser with a central wavelength of 1064.9 nm, and the full width at half maximum was measured to be approximately 2 nm by applying an Ocean Optics spectrometer (USB4000-VIS-NIR, Ocean Optics Inc, Dunedin, FL, USA), suggesting good monochromaticity of the Q-switched laser beam. The beam profiles of the CW and Q-switched lasers under a 2.5 W pump power are both shown in Figure 10b, obtained by applying a beam quality analyzer (BeamGate, Ophir-Spiricon, North Logan, UT, USA). As depicted, the beam shape of the Q-switched laser was modified compared to that of the CW laser; in particular, the central area of the laser beam was enlarged in Q-switched mode.

As a comparison, passively Q-switched laser results obtained when other 2-D heterojunction materials were used as an SA in solid-state lasers are summarized in Table 1. As presented, the stable ultrashort pulse generated in our work, down to 109 ns, was the narrowest among the 2-D heterojunction materials, and the peak power was also competitive. Compared with other laser resonators in Table 1, the cavity length of the resonator we designed is only an approximate 10 mm laser [49]. In addition, we coated a special optical film on the transparent surface of the laser crystal to effectively use the pump light in the laser crystal. Simultaneously, graphene oxide and Ti_2_CT_x_ have excellent optical properties [36,50]. Therefore, compared to other heterojunction materials in the solid-state laser experimental results, we obtained the shortest pulse width, while the average power and peak power were also competitive. In terms of pulsed laser applications, a short pulse width and high peak power have always been the parameters pursued by researchers [51]. Compared with other heterojunction materials, it has obtained better parameters in solid-state laser applications, such as pulse width, peak power, single pulse energy, and higher optical to optical conversion efficiency. These parameters are very meaningful for laser applications [52,53].

## 4. Conclusions

In conclusion, we proposed a facile method to synthesize an MXene–GO heterojunction in the liquid phase via a chemical reaction. The MXene exhibited an increased absorption signal and improved stability after being combined with GO material, compared to either the MXene material or GO material alone, and better photoelectric performance was achieved for the MXene–GO heterojunction. We demonstrated a passively Q-switched laser by using an MXene–GO heterojunction as an SA. A stable passively Q-switched pulse train can be obtained. The minimum observed pulse width was 109 ns, which is narrower than that of other Q-switched solid-state lasers using a 2-D heterojunction SA, and the corresponding repetition rate was 434.8 kHz. The maximum average output power was 344.2 mW, the maximum peak power was 7.25 W, and the maximum single-pulse energy was 0.79 μJ. Our experiments suggest the excellent performance of the MXene-based material in Q-switched solid-state lasers when combined with GO as an SA. To the best of our knowledge, this is the first report of a passively Q-switched Nd:YAG solid-state laser using MXene–GO as an SA. We believe that this will pave the way for designing 2-D heterojunction materials and further broaden their application.

## Figures and Tables

**Table 1:** Comparison of the performance of passively Q-switched lasers based on heterojunction materials as SAs.

SAs	Laser Type	*λ* (nm)	*τ* (ns)	*f*_rep_ (kHz)	*P*_ave_ (mW)	Efficiency	Pulse Energy (μJ)	Peak Power (W)	Refs
Te/BP	Nd:YAG	1064	329	126	312	/	2.48	7.53	[54]
	Tm:YAP	1980	250	83	1006		12.12	48.48	
	Er:YSGG	2800	163	151	330		1.92	11.78	
Sb_2_Te_3_-GO	Nd:GGG	1066	237	72	408	9.1%	5.67	23.92	[55]
GO-FONP	Nd:YVO4	1300	163	314	306	3.4%	0.974	5.98	[56]
Graphene/MoS_2_	Nd:YVO4	1064	180	640	190	2.8%	0.30	1.61	[57]
MoS_2_/Graphene	Tm:YAP	1942	473	105	553	8.0%	5.27	11.14	[58]
	Er:YSGG	2797	355	126	112	/	0.889	2.50	
MoS_2_/Graphene	Yb:GAB	1047	370	138.9	102	4.4%	0.734	1.98	[59]
SnSe_2_/MoS_2_	Nd:GGG	1061	526	61.2	370.8	8.24%	6.06	11.52	[60]
Ti_3_C_2_(OH)_2_/Ti_3_C_2_F_2_	Nd:YVO4	1064	130	508	300	12.2%	0.6	4.35	[61]
	Nd:YVO4	1340	390	195	480	18.1%	2.45	6.25	
Ti_2_CT_x_-GO	Nd:YAG	1064	109	434.8	344.2	9.3%	0.79	7.25	Our work

*λ*, central wavelength; *τ*, pulse width; *f*_rep_, repetition rate; *P*_ave_, average output power; efficiency, optical-optical conversion efficiency.
